# Editorial: Dismantling racial inequalities in higher education

**DOI:** 10.3389/fsoc.2024.1544431

**Published:** 2025-01-21

**Authors:** Carol Azumah Dennis, Judy Chandler

**Affiliations:** The Open University, Milton Keynes, United Kingdom

**Keywords:** racism in higher education, social change, anti-racist, higher education, dismantling racism

When our colleagues Dr. Jenny Douglas and Professor Marcia Wilson mooted the idea of this Research Topic—*Dismantling Racial Inequalities in Higher Education*, there were 27 black women professors^1^ in the UK. Five years later, that figure has increased by over 100%. In 2024—there were 66 Black Women Professors in UK Higher Education. This suggests something dramatic, giving cause for celebration, has happened. But while the figure has changed, the narrative remains the same. Racial disparity in UK Higher Education is not only experienced by the most established members of staff. All racialised bodies, from those seeking studentships to those undertaking undergraduate degrees witness similar levels of exclusion. Racial inequality is pervasive in its resonance and impact. But all of this is well documented, surrounded by decades of policy activity, from the ground-breaking Macpherson Report in 1999 to the more prosaic demands of the Race Equality Charter. Wearied by decades of talk about the dimensions and contours of racism, we wanted to move the discussion on.

We wanted to provide a practical toolkit, offering a road map outlining how racial discrimination in UK Higher Education could be dismantled. To disrupt Audre Lorde's saliant warning, we wanted to use the master's tools to dismantle the master's house.

This is not how the discussion went. While racial discrimination in HE is held firmly in place by headline figures–66 out of 23, 000 professors are black women—its contours are continually changing. The numbers change. The narrative stays the same. We may sound as if we are repeating ourselves by talking, yet again, about the existence, prevalence and nature of racial discrimination. But we have to keep talking about it because it keeps happening.

Tegama's paper reminds us just how substantial the dismantling task is, calling into question our understanding of its contours. There is, she argues, a malevolent interplay between theory, social mechanisms, and the university. An interplay which ultimately—through the color line (DuBois) or the abyssal line (de Sousa Santos 2014) legitimizes unequal power relations.

Ugiagbe-Green and Ernsting offer a generative pairing between wickedity and racism. They further argue that numbers do not and cannot tell the stories we need to hear. Using the statistical lens of QuantCrit, they expose the “awarding gap” as a metonymic of blame; one that erroneously imbues race with causative valiance. They urge an exploratory approach to understanding the racialised experiences of those caught in the snare of the “awarding gap”.

The danger of their approach is that it allows institutions the “*racial discrimination is elsewhere”* defense. It implies that the awarding gap exists because BME^2^ students enter the university less qualified and less ready to study, a deficit premised on the discrimination they have experienced *elsewhere*. Showunmi's paper forecloses this possibility. Her empirically grounded theorisation invokes “sophisticated racism”: systemic structures avowing to dismantle racism while disingenuously promoting inequitable practices. The sophisticated racist will talk the talk while doing everything they can to avoid walking the walk.

Green and Malcolm suggest a pathway toward dismantling racism in HE, by picking up the gauntlet of students-as-partners. Drawing on the philosophy of the Brazilian educator Paulo Freire, they propose anti-racist assessment practices as a liberatory mechanism. A great deal of weight is placed on anti-racist assessment. It is able to reshape the construction of a good student, while simultaneously producing graduates who are cognizant of their own privilege and positionality; emotionally literate; confident and competent in their… reflexivity and critical thinking; not only are they alert to injustice they are also minded to tackle it in and beyond their own lives.

Murphrey et al. include a necessary empirical basis for their suggested pathway through to dismantling racism. Evidencing identity or institutional barriers to student and professional progress, they map dismantling possibilities through careful navigation by outsiders or strategic removal by insiders.

One of the barriers faced by racialized others in the US is qualification. In contradistinction to Black Women in the UK, Hispanic women in the US are less qualified than their white counterparts. This systemically precludes them from leadership positions. Murphrey et al. distinguish between certification and competence. A degree does not confer capacity to leadership. This argument aligns with Bradley and Tillis' theory of organizational identity in sacred spaces of black education. Lived experience provides a fund of power and knowledge which may dismantle rigid structures holding discrimination in place.

It is left to Idowu to bring the collection together, combining empiricism, personal reflection and a dismantling toolkit. Her work exemplifies the power of invisible activism. The small quotidian, business as usual approach to getting things done. To getting the dismantling of racial discrimination in higher education done! She offers a compelling firsthand account of how she tackled the under-representation of BME postdoctoral researchers; and a later initiative in which she enhanced the visibility of BME academics in senior positions. With poetic simplicity her approach is one summed up with the three Ps of being positive, practical, and pragmatic.

This body of work ushers in further questions and potential research agendas for the academy to take forward in a mission to dismantle racial inequality in Higher Education. Firstly, the notion that a university should adopt a critical approach to its role within the societal machine. The university should exercise intentionality in what it chooses to legitimize or delegitimize and equip students to make sense of their experience of education, society, and contextualized social interactions, while also critiquing its own approaches to research and conceptualization of racial inequality. Secondly, complex narratives of lived racialised experience within the academy provide avenues for social change where they are suitably analyzed within the interconnected strands of context. The social environment is shifting and as such deeper analysis of this changing environment will strengthen research into the career paths of racially minorities academics and younger generations in their journeys into the workforce. Finally, for universities to affect social change, anti-racist approaches should sit alongside decolonization and diversification. It is only when taken as a complex whole that we can avoid these efforts leading to what Showunmi refers to as fictitious attainment.

## Footnotes

^1^It is worth noting a structural feature to the UK Higher Education. ‘Professorship' is a senior academic appointment to which after years of service senior lecturers can apply to be promoted conferring formal recognition of an outstanding career.

^2^In the UK, BME is an acronym for Black (and Brown) Minority Ethnic.

